# Management of blunt intraperitoneal bladder rupture: Case report and literature review

**DOI:** 10.1016/j.ijscr.2019.01.038

**Published:** 2019-02-01

**Authors:** Adel Elkbuli, John D. Ehrhardt, Shaikh Hai, Mark McKenney, Dessy Boneva

**Affiliations:** a Department of Surgery, Kendall Regional Medical Center, Miami, FL, United States; b University of South Florida, Tampa, FL, United States

**Keywords:** Urinary bladder rupture, CT cystography, Plain film cystography, Catheter-associated urinary tract infections

## Abstract

**Introduction:**

Urinary bladder ruptures are an uncommon injury, occurring in less than 1% of all blunt abdominal trauma. Extraperitoneal bladder ruptures are generally associated with pelvic fractures and usually managed nonoperatively. Conversely, intraperitoneal injuries are often caused by large compressive and shear forces produced during seatbelt injuries and almost invariably require surgical intervention.

**Presentation of case:**

A 29-year-old woman presented as a trauma alert after a motor vehicle collision with abdominal/flank pain and gross hematuria. Free intraperitoneal fluid was found on ultrasound and CT imaging. Exploratory laparotomy located an intraperitoneal rupture across the bladder dome. The patient recovered without complications, was discharged on postoperative day three, and continued bladder catheter care at home for an additional week until outpatient follow up and catheter removal.

**Discussion:**

As evidence for surgical management of bladder trauma continues to grow, clinical practice guidelines have been developed for trauma surgeons. Recent recommendations from the Eastern Association for the Surgery of Trauma appraise the evidence for cystography in the perioperative setting. Postoperative care is focused on preventing catheter-associated urinary tract infections in patients recovering from urotrauma in the critical care setting.

**Conclusion:**

We present a case of intraperitoneal bladder rupture in the setting of a blunt traumatic seatbelt injury. Our patient recovered uneventfully after surgical repair, a three-day hospitalization, and ten days with an indwelling bladder catheter.

## Highlights

• Urinary bladder rupture is uncommon, occurring in 0.36% of blunt abdominal trauma.
• Intraperitoneal ruptures are emergencies with >20% mortality when undiagnosed.
• CT and plain film cystography are the most sensitive and specific diagnostic imaging.
• Indwelling bladder catheters should remain in for at least 7 days postoperatively.

## 1 Introduction

Urinary bladder rupture entered the literature as early as 1826 when the *London Medical and Physical Journal* published two cases in the setting of blunt abdominal trauma [1]. One patient suffered multiple trauma after a collapsing load of construction rubble buried him. Another patient sustained a bladder rupture from a lower abdominal blow with a fence post during an intoxicated foot race. Today, bladder injuries remain relatively uncommon, representing only 0.36% of all blunt abdominal trauma admissions in a study of 15,168 patients [2].

Patients with bladder injury often present following motor vehicle collisions with suprapubic pain and gross hematuria. On examination, they can exhibit classic, yet nonspecific peritoneal signs that warrant further workup. Although bladder *contusion* is a common injury that may be treated non-operatively, full thickness bladder rupture should be considered in the differential diagnosis as a potential surgical emergency. Signs that raise the index of suspicion for bladder rupture include gross hematuria, pelvic fracture, acute kidney injury, and little to no urine output with catheterization [3].

Bladder ruptures can be intraperitoneal, extraperitoneal, or both. Intraperitoneal ruptures which comprises 15% of all bladder ruptures are associated with severe blunt force trauma to a distended bladder. These injuries often occur across the bladder dome where the wall is mobile, attenuated and predisposed to balloon out. Fortunately, intraperitoneal lacerations are amenable to surgical repair with success rates at 100 percent in several case series [4,5]. Extraperitoneal ruptures are more common (80% of cases), generally occur in the setting of pelvic fracture, and involve the anterolateral bladder wall, trigone, and bladder neck. A minority of patients have combined intraperitoneal and extraperitoneal injury (5% of cases). Although some complex extraperitoneal ruptures are surgically repaired, the literature currently suggests that most extraperitoneal ruptures can be non-operatively managed [6].

Herein, we present a case of isolated intraperitoneal bladder rupture following a motor vehicle collision. Surgical management will be discussed in context of recent clinical practice guidelines for bladder rupture from the Eastern Association for the Surgery of Trauma [7]. This case is reported with consideration to the SCARE criteria [8].

## 2 Presentation of case

A 29-year-old woman presented to the emergency department following a motor vehicle collision in which she was the seatbelt-restrained driver. Airbags deployed when her vehicle hit a roadside tree at a high speed. Emergency medical services arrived at the scene and recommended transport to a Trauma Center, but she chose to go home against their recommendation. Over the course of the next few hours, she developed severe lower abdominal pain, gross hematuria, and called for ambulance transport.

**Fig. 1:**
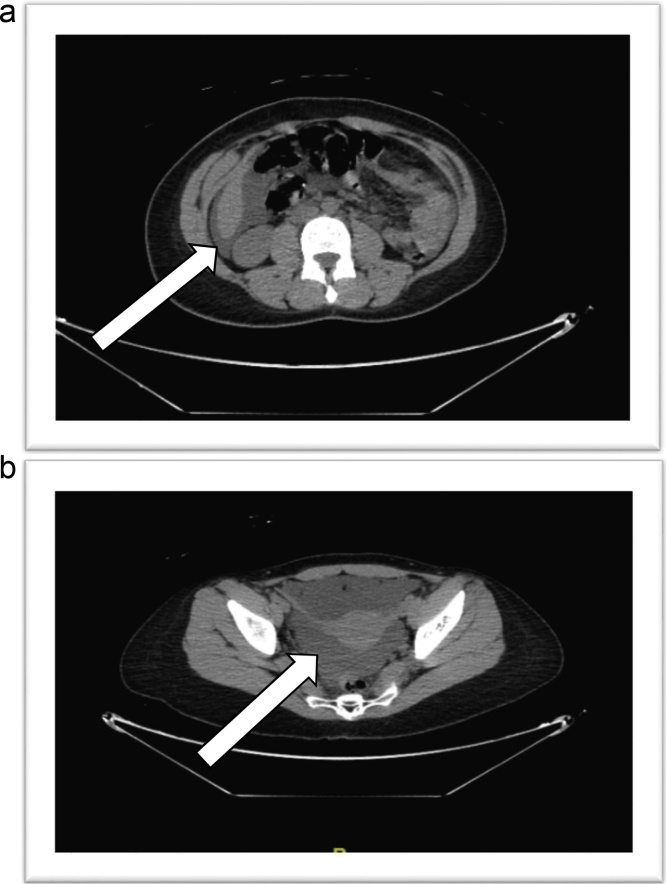
**A:** CT abdomen showing simple fluid density (urine) in perihepatic space and hepatorenal fossa. **B:** CT pelvis showing simple fluid density (urine) surrounding the uterus.

Upon arrival as a Trauma Alert, she was in severe abdominal and flank pain. She appeared lethargic and confused, but had not experienced head trauma during the collision. She was afebrile but tachycardic, with normal blood pressure. Her abdomen was distended and guarded with rebound tenderness. There was frank blood at the urethral orifice and introduction of a bladder catheter yielded no urine. Plain radiographs of the pelvis revealed no evidence of fracture. Laboratory data were significant for a hemoglobin of 12.0 mg/dL, leukocytosis of 15.1 x 10^3^ WBC/μL, and elevated serum creatinine of 1.57 mg/dL. Non-contrast CT abdomen and pelvis showed no evidence of acute trauma, but revealed a moderate amount of simple fluid density ascites within the abdomen and pelvis of unknown etiology (Fig. 1). At this time, diagnoses included blunt abdominal injury with free intraperitoneal fluid, peritonitis, acute kidney injury, metabolic acidosis, and dehydration.

**Fig. 2:**
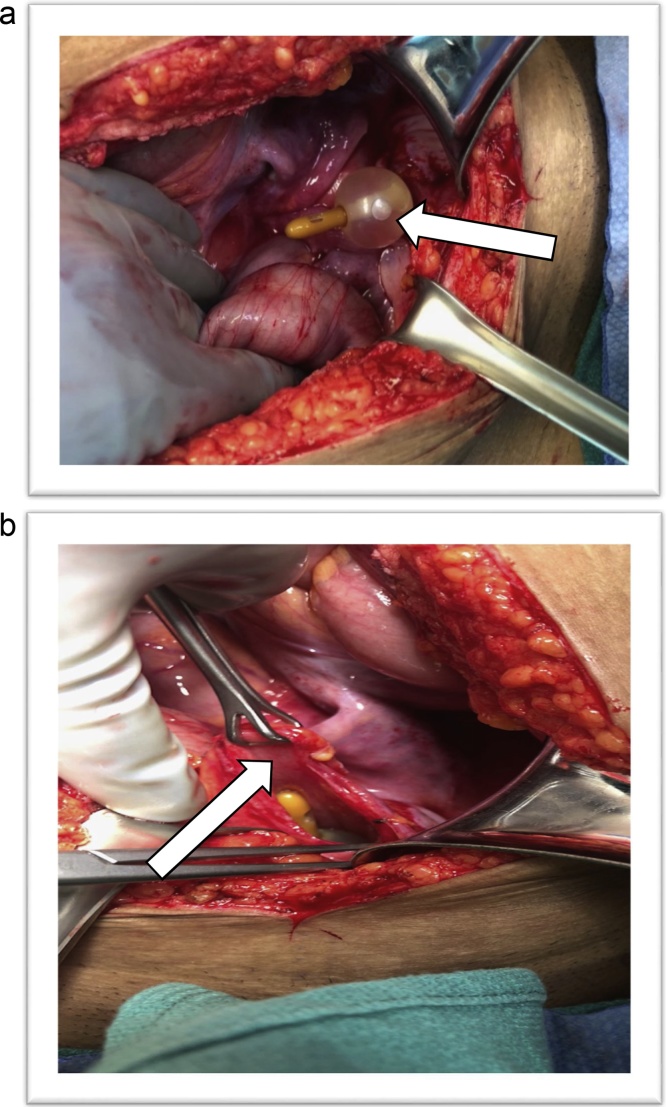
**A:** Intraoperative view of low midline laparotomy showing ruptured bladder dome with bladder catheter and inflated balloon. **B:** Ruptured bladder wall held with Babcock forceps.

Trauma surgery then performed an emergent exploratory laparotomy and found a full-thickness rupture across the bladder dome (Fig. 2). Approximately one liter of clear urine was drained from the abdomen. The bladder was repaired in two layers with absorbable sutures and a bladder catheter was left in place. The bladder repair was then tested intraoperatively by filling the bladder with fluid via the bladder catheter until the bladder distended and no leak was observed.

**Fig. 3:**
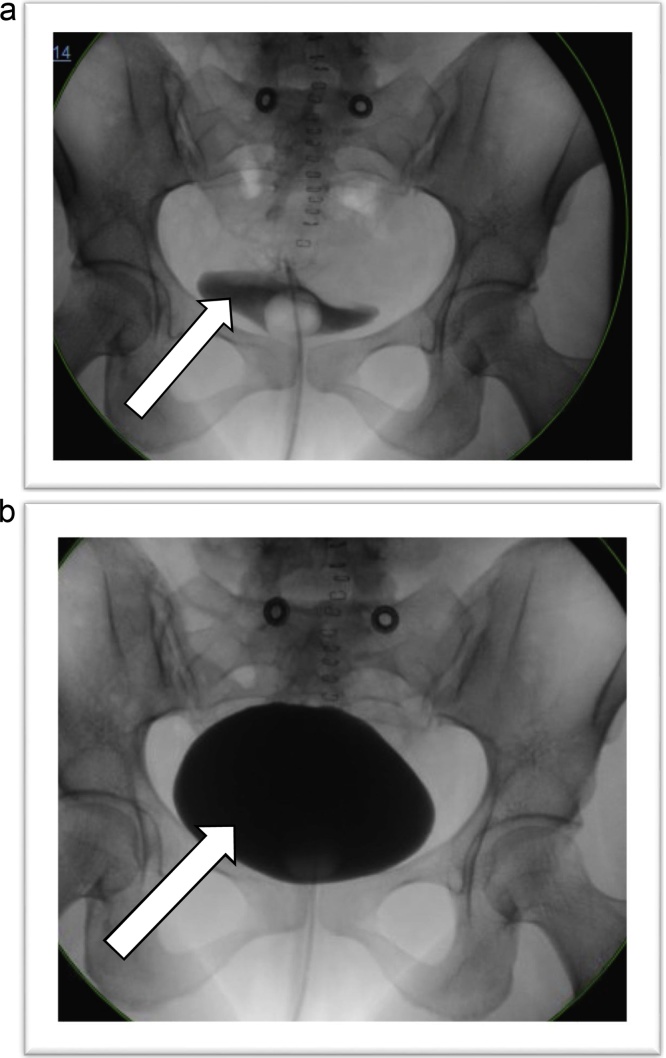
**A:** Postoperative cystogram of bladder filling with contrast and no leak. **B:** Postoperative cystogram demonstrating full bladder with no leakage.

On postoperative day one, she was transferred from the ICU to the medical/surgical unit in stable condition. Laboratory data showed her leukocytosis normalized as did her serum creatinine. She continued to recover well, advancing to a regular diet on postoperative day two with her pain well controlled. By postoperative day three, hematuria resolved, and cystography showed no leakage (Fig. 3). She was discharged that day with instructions to keep the bladder catheter in place for seven more days. Follow up in the outpatient trauma clinic the following week, showed she was without complications; and the indwelling catheter was removed without incident.

## 3 Discussion

Our patient was a seatbelt-restrained driver who suffered an intraperitoneal bladder rupture in the absence of pelvic fracture. Intraperitoneal injuries generally follow blunt force compression of a large, distended bladder positioned at the posterior aspect of the ventral abdominal wall and superior to the protection afforded by the bony pelvis. With respect to motor vehicle collisions, deceleration against seatbelt restraints represent a common injury mechanism. In these cases, the weak and mobile bladder dome ruptures, and remaining forces distribute throughout the abdomen, often causing multiorgan injury.

Management of intraperitoneal bladder rupture hinges on early recognition and surgical intervention. While extraperitoneal ruptures can generally be managed non-operatively with a bladder catheter, guidelines from both the American Urologic Association and the Eastern Association for the Surgery of Trauma advocate for surgical repair of intraperitoneal lacerations [6,9]. Free urine in the abdomen and pelvis leads to acute renal failure and sepsis, which is responsible for mortality rates over 20% when not promptly identified [10]. Our patient exhibited acute kidney injury and neurologic manifestations within hours of injury.

Several imaging studies can provide evidence to raise the index of suspicion for bladder rupture. Sonographic scans can identify free fluid in the pelvis [11], but are unreliable in distinguishing between fluids like urine, blood or succus. Non-contrast CT scans can identify simple fluid ascites, but often cannot identify a laceration on the bladder itself, as in our case and others in the literature [12]. Both plain film cystography and CT cystography are highly sensitive and specific studies available for the diagnosis or exclusion of bladder rupture [13,14]. CT cystography may be preferred in the setting of trauma where spine boards and fragments of pelvic fractures may mask bladder injury under fluoroscopy. Nonetheless, the additional time needed, summative radiation exposure, and financial burden of CT cystography may not be appropriate in unstable patients and those with acute abdomen and free intraperitoneal fluid who already require laparotomy for other concurrent intra-abdominal injuries.

Operative repair can be performed by trauma surgery or urology. One retrospective study from South Africa noted that their trauma service repaired all intraperitoneal bladder ruptures except those complicated by concomitant extraperitoneal rupture and complex pelvic injury, in which case they consulted a urologic surgeon for support [15]. Laparotomy is generally preferred when there is concern for multi-organ trauma, but reports and case series have demonstrated excellent outcomes with laparoscopic repair [16,17]. One or two-layered closure of the dome is accomplished with absorbable sutures. Permanent sutures represent a potential nidus for calcium deposition and future bladder stone formation [18].

Postoperative cystography can identify leaks in the repaired bladder wall, but studies have shown it is often unnecessary [5,19]. The Eastern Association for the Surgery of Trauma recently synthesized the body of evidence and produced recommendations for postoperative cystography based on whether the intraperitoneal injury was simple (one full-thickness tear) versus complex (accompanied by other urinary bladder lesions). For simple intraperitoneal ruptures, they recommend against cystography on the premise that only one leak in 1000 patients would be detected with the remainder 999 patients undergoing unnecessary testing. Conversely, they support cystography after *complex* ruptures for its ability to diagnose leaks in 87 out of 1000 patients, thereby avoiding morbidity associated with undiagnosed postoperative leakage. These recommendations were considered strong but admittedly based on low-quality evidence [6].

Postoperative hospital care is focused on monitoring urine output and maintenance of an unclogged and free flowing indwelling bladder catheter to prevent catheter-associated urinary tract infections (CAUTI’s). On postoperative day one the patient’s urine output was over 4 liters. This is likely due to “post obstructive polyuria” as a normal physiologic response to eliminate volume and solutes that accumulated. Bladder catheter maintenance is crucial to prevent CAUTI’s, especially with the highly susceptible patient population seen in trauma and critical care settings [20]. Antibiotic prophylaxis can reduce the incidence of CAUTI’s throughout the duration of an indwelling catheter [21], but further precautions are necessary. Nursing education for proper bladder bag placement and twice-daily perineal care reduces CAUTI’s as much as 70% in trauma patients [22]. On discharge, patients are instructed to continue these measures until one-week outpatient follow up.

## 4 Conclusion

We present a case of a 29-year-old woman with intraperitoneal bladder rupture in the setting of a blunt traumatic seatbelt injury. Our patient recovered uneventfully after surgical repair, a three-day hospitalization, and ten days with an indwelling bladder catheter. Recently developed clinical practice guidelines reaffirm the significance of surgical repair for intraperitoneal injuries and appraise the evidence for cystography in the perioperative setting. Postoperative care is focused on preventing catheter-associated urinary tract infections.

## Conflicts of interest

None.

## Sources of funding

None.

## Ethical approval

This report was conducted in compliance with ethical standards.

## Consent

Written and signed patient consent was obtained.

## Author contribution

Adel Elkbuli, Dessy Boneva, John D. Ehrhardt Jr – Conception of study, acquisition of data, analysis and interpretation of data.

Adel Elkbuli, Dessy Boneva, John D. Ehrhardt Jr, Shaikh Hai - Drafting the article.

Dessy Boneva, Mark McKenney – Management of case.

Adel Elkbuli, John D. Ehrhardt Jr, Dessy Boneva, Shaikh Hai, Mark McKenney – Critical revision of article and final approval of the version to be submitted.

## Registration of research studies

This is a case report study.

## Guarantor

Dessy Boneva.

Mark McKenney.

Shaikh Hai.

## Provenance and peer review

Not commissioned, externally peer-reviewed.
